# Self-templated synthesis of novel carbon nanoarchitectures for efficient electrocatalysis

**DOI:** 10.1038/srep28049

**Published:** 2016-06-15

**Authors:** Xi-Lin Wu, Tao Wen, Hong-Li Guo, Shoujie Liu, Xiangke Wang, An-Wu Xu, Markus Mezger

**Affiliations:** 1 School of Nuclear Science and Technology, Division of Nanomaterials & Chemistry, University of Science and Technology of China, Hefei 230026, P.R. China; 2 School of Environment and Chemical Engineering, North China Electric Power University, Beijing 102206, China; 3 College of Geography and Environmental Science, Zhejiang Normal University, Jinhua 321004, China; 4 Max Plank Institute for Polymer Research, Ackermannweg 10, 55128, Mainz, Germany; 5 Key Laboratory of Novel Thin Film Solar Cells, Institute of Plasma Physics, Chinese Academy of Sciences, Hefei 230031, P.R. China; 6 Institute of Physics, Johannes Gutenberg University Mainz, 55128 Mainz, Germany

## Abstract

The cost-efficient large-scale production of novel carbon nanostructure with high performance is still a challenge, restricting their applications in catalysis. Herein, we present a low-cost one-pot and one-step approach for the synthesis of both N-doped graphene (NG) and N-doped carbon nanotubes (NCNTs) from self-templated organic nanoplates. By varying the FeCl_3_ concentration in the precursor, we can control the formation of graphene or CNTs. To the best of our knowledge, this is the first example for the controllable synthesis of graphene or CNTs by varying the precursors’ compositions. This provides a simple and cost-effective route for the large-scale production of both NG and NCNTs for applications in catalysis. By example, we show how these unique structured nanocarbons can be applied in electrocatalysis for oxygen reduction reaction (ORR). The obtained NG and NCNTs show excellent ORR activities with long-term stability under alkaline conditions. The unique porous nanostructure, abundant defects, homogeneous N-doping and high N-content in the NG and NCNTs can provide abundant active sites, leading to the excellent ORR performance. This research not only displayed a simple and cost-effective approach for the large-scale production of novel carbon nanoarchitectures, but also revealed the exceptional application potential of these nanocarbons for electrocatalysis.

Nanocarbons are a large class of carbonaceous materials with tailored nanoscale dimension and unique structure dependent properties^1^,^2^. Graphene and carbon nanotubes (CNTs) are two classic nanocarbons. Graphene is composed of sp^2^ carbons arranged into a two-dimensional (2D) carbon sheet, while CNTs can be considered as that the 2D carbon sheet rolled up into a seamless cylinder with a nanoscale diameter. As representative 1D and 2D nanomaterials, CNTs and graphene have triggered extensive studies in both theoretical and practical aspects. Both graphene and CNTs hold many unprecedented intrinsic properties such as high Young’s modulus, large tensile strength and high electric and thermal conductivity^2^,^3^. These intrinsic properties are further coupled with lightweight, high surface area and excellent chemical stability, offering them potential applications in many fields such as electronics, biomedicine, catalysts, composite functional materials and energy storage and conversion^1^,^2^,^3^,^4^. Many methods have been developed for the synthesis of graphene and CNTs, including chemical vapor deposition (CVD), arc discharge and chemical or electrochemical exfoliation^1^,^4^. Although the CVD method is successfully to produce large-area single or few layer graphene, it inevitably requires complex facilities and harsh operation conditions^1^,^2^. While the arc discharge method holds the disadvantages of using specialized facilities and high-cost. Graphene prepared by CVD or arc discharge is not suitable for catalysis applications due to its difficulties in scale up production and high-cost^5^. The chemical or electrochemical exfoliation methods usually suffer from using toxic chemicals, numerous steps, and low yields^4^. Thus, large-scale production of both graphene and CNTs with low-cost and high performance is still challenge.

The oxygen reduction reaction (ORR) is considered as a key process in fuel cells and metal-air batteries^6^,^7^. The sluggish ORR is one of the main obstacles to achieve high efficiency in fuel cells. Currently, platinum-based materials are the predominantly used catalysts for ORR, however, their scarcity and high-cost restrict fuel cells for large-scale commercial application^6^. Many efforts have been devoted to developing alternative catalysts with low-cost and high performance in order to replace the commercial Pt/C catalyst. Among the many alternatives, nanocarbon based metal-free or noble metal-free catalysts are recognized as the most promising candidates for ORR because of their low-cost, good stability and sustainability. As two of the most studied nannocarbons, CNTs and graphene have drawn extensive attentions due to their extraordinary intrinsic properties. Recently, heteroatom doped CNTs or graphene have been demonstrated to be promising precious-metal-free catalysts for ORR. For example, B-doped CNTs^8^, N-doped CNTs^9^, and S, N co-doped CNTs^10^ have been synthesized and found to exhibit an enhanced catalytic activity for ORR as compared to the pristine CNTs. Similarly, graphene doped with various elements such as B, N, P, O and S was also prepared and showed improved ORR catalytic activity^11^,^12^,^13^. Among the many doping elements, N is the most widely introduced heteroatom due to its large electronegativity (3.04)^10^. Introduction of N atoms into the carbon networks of CNTs or graphene can change their electronic structure and create charged sites, which are favorable to the molecular adsorption of O_2_ and its reduction^12^.

Herein, we have developed a cost-effective one-pot approach for the synthesis of N-doped graphene (NG) and N-doped CNTs (NCNTs). A self-templated polymer and the mixture of FeCl_3_ with the polymer were utilized as precursors for the preparation of the NG and NCNTs, respectively. The obtained NG possesses a well-defined porous structure with large surface area, which is favorable to the mass transfer from both the electrolyte ions to electrode and the electrons to active sites^14^. Moreover, the high N content and large surface area of the NG can provide more exposed active sites, which are beneficial to the adsorption of O_2_ and its reduction, leading to high ORR activity. Similarly, the obtained NCNTs also show excellent ORR activity.

## Results and Discussion

### Materials Characterization

**Figure 1:**
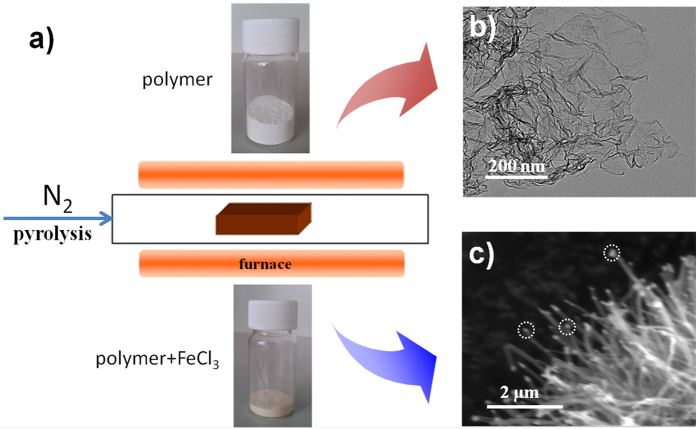
(**a**) Schematic illustration of the experimental set-up for the one-pot synthesis of N-doped graphene and N-doped CNTs. (**b**) TEM image of the obtained N-doped graphene. (**c**) SEM image of the obtained N-doped CNTs.

**Figure 2:**
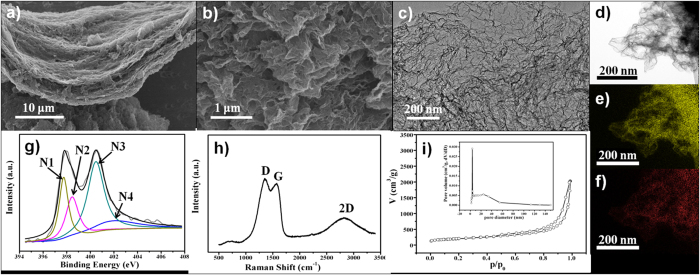
(**a**,**b**) SEM images and (**c**) TEM image of the NG-800. (**d**) HAADF–STEM image of the NG-800, and the the corresponding elemental mapping image of (**e**) C and (**f**) N. (**g**) High resolution N 1s XPS spectra of the NG-800. The N 1s peak is deconvoluted into four peaks located at 397.7 (N1), 398.5 (N2), 400.5 (N3) and 401.9 (N4) eV. (**h**) Raman spectrum of the NG-800. (**i**) N_2_ sorption isotherms of the NG-800. Insets show the pore size distribution of the NG-800 calculated by the BJH method.

The synthetic process was accomplished through a convenient set-up by annealing the precursors at various temperatures (700–900 °C) under N_2_ atmosphere (Fig. 1a). After the annealing process, porous graphene (Fig. 1b) or CNTs forests were obtained (Fig. 1c). Scanning electron microscopy (SEM) image (Fig. 2a) shows the multilayered morphology of the NG-800 at a large scale and the NG nanosheets are stacked together. Enlarged SEM image (Fig. 2b) reveals the porous structure of NG with many folds and wrinkles. The there-dimensional (3D) corrugated structure of the NG sheets was also demonstrated by the transmission electron microscopy (TEM) image (Fig. 2c). Such a 3D structure can provide more contact interfaces, thereby providing more exposed active surface. From the high-resolution TEM (HR-TEM) image (Figure S1), it can be seen that there are many “patched” graphene nanodomains. These carbons can be considered as polycrystalline graphene nanoplatelets^15^,^16^. The high-angle annular dark-field scanning TEM (HAADF-STEM) images (Fig. 2d–f) display the homogeneous distribution of N atoms all over the NG nanosheets. Further elemental analysis shows the high N content in the NG-800 with a mass percentage of 11.7%. The N-enrichment precursors, that is, melamine, cyanuric acid and polyethyleneimine (PEI), are responsible for the high N content in the obtained NG. The X-ray photoelectron spectroscopy (XPS) analysis was applied to verify the surface elements of the NG-800. The presence of N element in the NG-800 is observed at a binding energy of 400.0 eV, as shown in the XPS survey spectra in Figure S2. The high resolution N 1s XPS spectrum (Fig. 2g) is deconvoluted into four peaks located at 397.7, 398.5, 400.5 and 401.9 eV, coresponding to the pyridinic N (N1, 20.9%), pyrrolic N (N2, 19.2%), graphitic N (N3, 43.2%) and oxygenated N (N4, 16.6%), respectively. Among the four types of the N species, the graphitic N is the dominant species, which has been proven to be a key factor to achieve high performance in electrocatalysis^17^. Raman spectrum (Fig. 2h) reveals the characteristic peaks at 1352, 1575, 2820 cm^–1^ corresponding to the D, G and 2D band, respectively. The broad D band is indicative of the presence of disorder and defects in the graphene, while the G band is related to the in-plane vibration of the sp^2^ carbons^18^. The integrate intensity of I_D_/I_G_ ratio is calculated to be about 2.3, which also confirms the presence of defects. And these defects may provide more exposed active sites, which is beneficial to the molecular adsorption and ion-transportation in electrocatalysis. The N_2_ adsorption-desorption measurements (Fig. 2i) demonstrate a high Brunauer–Emmett–Teller (BET) surface area of the NG-800 (710 m^2^/g) with a large pore volume (1.84 cm^3^/g). The pore size distribution shows the pores are mainly located in the range from several nanometers to a hundred nanometer with a sharp peak located at 3.7 nm. The type-IV isotherm displays the obvious adsorption at high relative pressures (0.5–1.0 P/P_o_), which also confirms the existence of lots of mesopores^19^. The obtained NG-800 possesses a well-defined porous structure with large surface area, which is favorable to the mass transfer from both the electrolyte ions to electrode and the electrons to active sites in electrocatalysis^14^. The introduced N atoms in combination with the well-organized porosity and high surface area of the NG-800 may offer a great potential to achieve high performance in electrocatalysis.

Contrast experiment was carried out by pyrolysising of self-assembled MCA polymer (details for the preparation of MCA is shown in supporting infromation) without PEI under the same conditions. Interestingly, we found that no solid was obtained. As previous report, the annealing of MCA polymer at 550 °C could lead to g-C_3_N_4_^20^, while g-C_3_N_4_ will be totally decomposed at temperature above 700 °C, thereby g-C_3_N_4_ can act as template to form 2D carbon structures^18^. The adsorbed PEI on the surface of MCA is carbonized and transformed into graphene nanosheets on account of the confinement by the layered g-C_3_N_4_^14^. Thus, the growth of the porous graphene occurs via a self-templated process by decomposing and carburnization of the PEI-MCA nanoplates. As compared to other methods such as hard-template^13^ or chemical doping of graphene oxide^11^,^12^, this self-templated method holds advantages of using low-cost raw materials and one-step process, which makes this strategy more promising for the application in catalysis.

**Figure 3:**
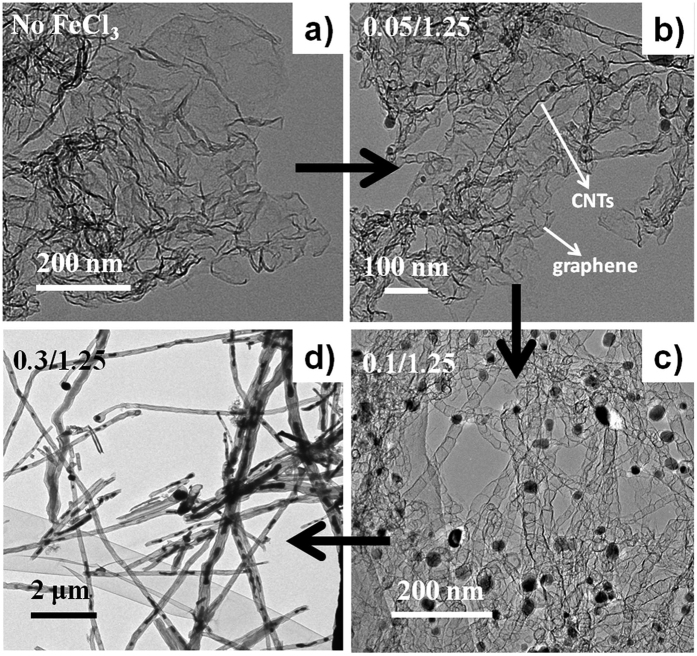
TEM images of the products obtained by varying the weight ratio of FeCl_3_/PEI-MCA in the precursor. (**a**) No FeCl_3_ was added, (**b**–**d**) weight ratio of FeCl_3_/PEI-MCA are 0.05/1.25, 0.1/1.25 and 0.3/1.25, respectively.

For the preparation of NCNTs, the mixture of the PEI-MCA nanoplates with FeCl_3_ were applied as precursors and subject to pyrolysis at 800 °C under N_2_ atomosphere. The product is denoted as NCNTs-800. From the SEM image (Fig. 1c), the CNTs forests with white dots at tips of the CNTs are observed. The black dots correspond to the iron nanoparticles which were produced by the carbothermal reduction of Fe^3+^ ions during the pyrolysis process. The transformation of graphene to CNTs is observed by varying the ratio of FeCl_3_/PEI-MCA in the precursors. As can be seen from Fig. 3, the morphology of the product changes from graphene (Fig. 3a) to graphene/CNTs composites (Fig. 3b), then to bamboo-like CNTs (Fig. 3c), and finally to long and thick CNTs (Fig. 3d) upon changing the weight ratio of FeCl_3_/PEI-MCA from 0 to 0.3/1.25. These results suggest the growth of CNTs could be attributed to the curling of the two-dimensional graphene catalyzed by the iron nanoparticles during the pyrolysis process.

**Figure 4:**
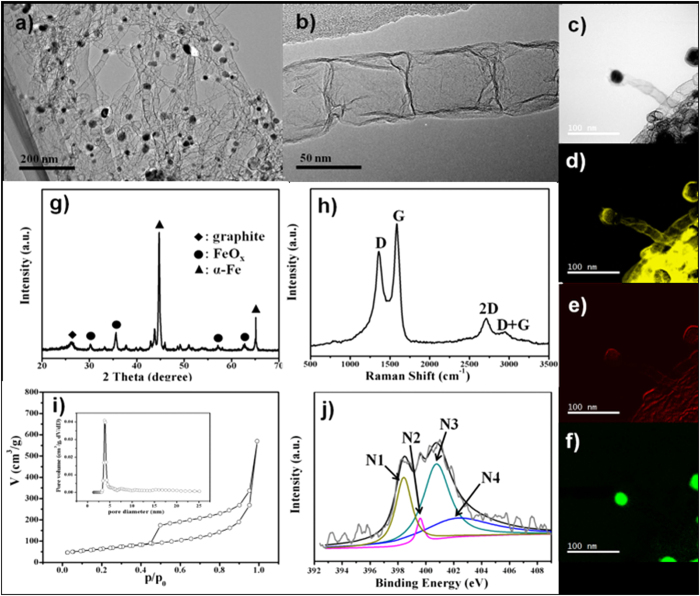
(**a**,**b**) TEM image of the NCNTs-800. (**c**) HAADF–STEM image of the NCNTs-800, and the the corresponding elemental mapping image of (**d**) C, (**e**) N and (**f**) Fe. (**g**) XRD pattern of the NCNTs-800. (**h**) Raman spectrum of the NCNTs-800. (**i**) N_2_ sorption isotherms of the NCNTs-800. Insets show the pore size distribution of the NCNTS-800 calculated by the BJH method (**j**) high resolution N 1s XPS spectra of the HCl etched NCNTs-800. The N 1s peaks is deconvoluted into four peaks located at 398.4 (N1), 399.6 (N2), 400.8 (N3) and 402.2 (N4) eV.

From the TEM image of NCNTs-800 (Fig. 4a), it is observed that bamboo-like CNTs entangle together with iron nanoparticles inside the tubes. The enlarged TEM image (Fig. 4b) further demonstrates the bamboo-like structure of the NCNTs-800 with a diameter of ~50 nm. The formation of the bamboo-like structure suggests the N-doped CNTs were successfully prepared^10^. HAADF–STEM image reveals the iron nanoparticles are located at the tip of the CNTs, and the corresponding elemental mapping images confirm the homegeneous distribution of C and N elements over the entire CNTs (Fig. 4c–f). From Fig. 4g, the XRD pattern demonstrates the NCNTs-800 sample is composed of graphite, FeO_x_ and α-Fe^21^. During the pyrolysis process, most of the Fe^3+^ ions were reduced into Fe nanoparticles, which catalyze the growth of the bamboo-like CNTs. Raman spectrum (Fig. 4h) of the NCNTs-800 clearly displays the characaeristic peaks at 398.4, 399.6, 400.8 and 402.2 eV, corresponding to the D, G, 2D and D+G band, respectively. The strong G band is indicative of the graphite structure of the CNTs. The integrate intensity of I_G_/I_D_ ratio are calculated to be about 0.7, indicating the presence of defects^22^. The N_2_ adsorption-desorption results (Fig. 4i) display a hysteresis loop at high relative pressures (0.5–1.0 P/Po), corresponding to the type-IV isotherm, confirming the existence of mesoporous in NCNTs-800^19^. The BET surface area of the NCNTs-800 was measured to be 225 m^2^/g. The pore size distribution further demonstrates the pores are mainly located at the mesoporous region with a sharp peak at about 3.9 nm. For XPS analysis and electrochemical measurements, the NCNTs-800 was pre-treated by 6 M HCl to remove the inactive Fe species. From the XPS survey spectrum (Figure S2), the N peak at binding energy of 400.0 eV is observed. While the absence of Fe peak suggests the inactive surface Fe species was successfully etched by HCl. The N content in NCNTs-800 is about 2.88 atom%, which is much lower than that in NG-800 (14.89 atom%). The high resolution N 1s XPS spectrum (Fig. 4j) is deconvoluted into four peaks located at 398.4, 399.6, 400.8 and 402.2 eV, coresponding to the pyridinic N (N1, 23.8%), pyrrolic N (N2, 3.7%), graphitic N (N3, 43.3%) and oxygenated N (N4, 29.1%), respectively. The high surface area combined with the uniform N-doping of the NCNTs-800 may lead to high performance in electrocatalysis.

### Electrochemical activity and durability

**Figure 5:**
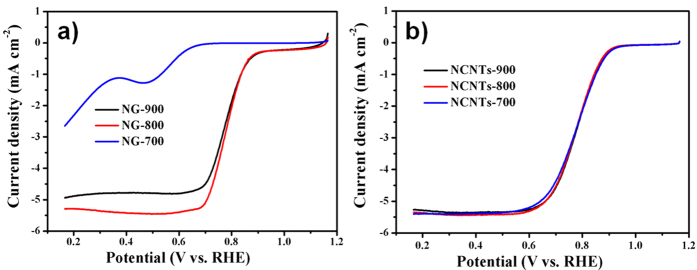
RDE voltammograms of the (**a**) NG samples pyrolyzed at temperature of 700, 800 and 900 °C (denoted as NG-700 NG-800 and NG-900, respectively), and the (**b**) NCNTs samples pyrolyzed at temperature of 700, 800 and 900 °C (denoted as NCNTs-700 NCNTs-800 and NCNTs-900, respectively).

The electrocatalytic properties of the NG and NCNTs samples for ORR were systematically investigated by using cyclic volta-mmogram (CV) measurements in the Ar or O_2_ saturated KOH solution. To find out the optimum pyrolysis temperatures for our catalysts, rotating disk electrode (RDE) experiments were conducted to determine the ORR activity of the samples prepared at various temperatures (700, 800 and 900 °C) (Fig. 5). The best ORR activity of the NG was achieved at 800 °C, while the ORR activities of the NCNTs samples prepared at different temperatures do not show much differences. The different ORR activities of the NG samples could be due to the different surface area, electric conductivity and active site density realized at various temperature^17^. As shown in Figure S3a, featureless double-layer capacity current was observed in CV curve of the NG-800 in N_2_ saturated electrolyte. While in O_2_ saturated electrolyte, a well- defined peak centered at about 0.66 V appears, suggesting a pronounced ORR activity of the NG-800. For further determing the ORR activity of the NG-800, RDE experiments were performed at rotating speed of 1600 rpm and voltage scanning rate of 10 mV s^−1^ in O_2_ saturated 0.1 M KOH solution. The ORR polarization plots (Figure S3b) show the onset potential (*E*_*onset*_) of the NG-800 (0.92 V) is about 51 mV lower than that of commercial Pt/C (0.97 V), and half-wave potential (*E*_*1/2*_) of the NG-800 is only 28 mV more negative than that of Pt/C. The limiting current density (*j*_*L*_) of the NG-800 catalyst is 5.29 mA cm^−2^ at a rotating speed of 1600 rpm which is comparable to that of Pt/C (5.49 mA cm^−2^). These electrochemical values are close to those of commercial Pt/C and higher than those of previous reported N-graphene^13^ (*E*_*onset*_ = 0.895 V, *j*_*L*_ ~ 4.0 mA cm^−2^) prepared by a hard template method. The electron transfer number of the NG-800 catalyst for the reduction of oxygen was further investigated by using the rotating ring–disk electrode (RRDE) technique (Figure S3c). The electron transfer number (Figure S3d) calculated from the RRDE data is in the range of 3.55–3.88 at the potential window of 0.17 to 0.91 V, indicating the ORR process mainly occurs via a four-electron path. The ORR durability of the catalyst was investigated by using continuous CV measurments within a potential range of −0.8 to 0.2 V (vs. Ag/AgCl) in O_2_ saturated 0.1 M KOH solution. After 10000 CV cycles, the *E*_*1/2*_ of the NG-800 shifted to negative direction for only 16.0 mV (Figure S4c), much lower than that of Pt/C (about 65.0 mV, Figure S4b), suggesting the distinguished durability of the NG-800. Moreover, the metal-free NG catalysts can be synthesised at a large scale by a simple and cost-effective self-templated route, indicating that the NG-800 is more sustainable than the expensive commercial Pt/C catalysts.

**Figure 6:**
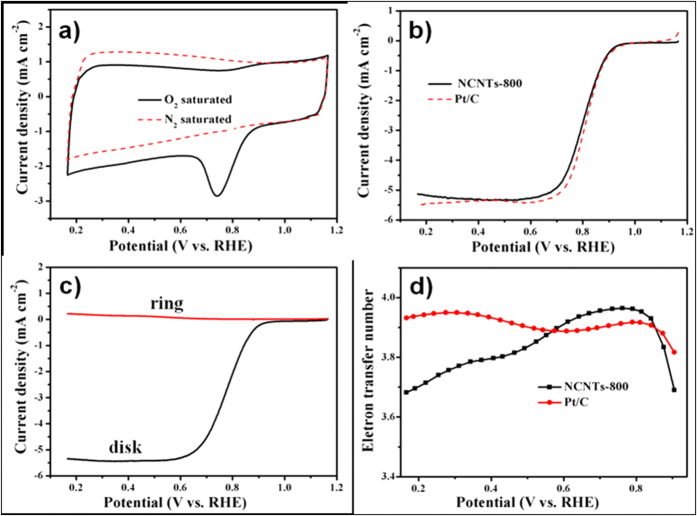
(**a**) Cyclic voltammograms (CV) of the NCNTs-800 in O_2_ and N_2_ saturated 0.1 M KOH solution. (**b**) Rotating disk electrode (RDE) voltammograms of the NCNTs-800 and Pt/C at rotating speed of 1600 rpm and voltage scanning rate of 10 mV s^−1^ in O_2_ saturated 0.1 M KOH solution, (**c**) rotating ring–disk electrode (RRDE) voltammograms of the NCNTs-800 at rotating speed of 1600 rpm and voltage scanning rate of 10 mV s^−1^ in O_2_ saturated 0.1 M KOH and (**d**) the corresponding electron transfer number.

Similarly, the obtained NCNTs-800 samples also displayed a pronounced ORR activity in alkaline medium. As show in Fig. 6a, the NCNTs-800 exhibits a well-defined ORR peak at potential of 0.74 V in O_2_ saturated electrolyte. The absence of ORR peak of the CV plot in the N_2_ saturated electrolyte further confirms the ORR activity of the NCNTs-800. RDE voltammograms (Fig. 6b) show the *E*_*onset*_ of the NCNTs-800 is 0.96 V, which is much close to that of Pt/C (0.98 V). *E*_*1/2*_ of the NCNTs-800 is about 0.80 V, which is a little lower than that of Pt/C (0.81 V). These electrochemical values are very close to those of Pt/C, suggesting the high ORR activity of the NCNTs-800 in alkaline medium. RRDE voltammograms exhibit the ring and disk currents of the NCNTs-800 at a rotating speed of 1600 rpm and voltage scanning rate of 10 mV s^−1^. Figure 6c shows the disk and ring currents measured at 1600 rpm in O_2_ saturated 0.1 M KOH. The H_2_O_2_ yield calculated from the RRDE data is shown in Figure S4a. The H_2_O_2_ yield of the NCNTs-800 is below 15.9% over the measured potential range and lower than 5% at potential range of 0.6–0.8 V, suggesting the four-electron path was dominated in the ORR process. Especially, the H_2_O_2_ yield of the NCNTs-800 is lower than that of Pt/C at high potential (0.6–0.8 V). The plots of the electron-transfer number further demonstrate the four-electron process of the NCNTs-800 (Fig. 6d). The NCNTs-800 sample shows predominant durability with only 8 mV of the *E*_*1/2*_ shift after 10000 CV cycles (Figure S4d), implying a much better durability than Pt/C. The results demonstrate the obtained NCNTs can be suitable non-noble metal catalyst for oxygen reduction reaction.

According to the above structural and compositional characterizations, the high ORR activity of the NG-800 and NCNTs-800 could be attributed to their unique porous structure, large surface area, high content of N species and homogeneously N distribution. And those characters not only provide many exposed actives sites but also allow the sustained and stable transport of both electrolyte ions and electrons on the surface of catalysts, leading to excellent ORR performance. The ORR activities of the NG-800 and NCNTs-800 were further investigated in acid medium (Figure S5). The NG-800 and NCNTs-800 showed comparable *E*_*onset*_ at about 0.70 and 0.78 V, respectively. Although these activitites are still inferior to the Pt/C (*E*_*onset*_ = 0.86 V).

## Conclusion

In conclusion, a simple one-pot and one-step approach was developed for the snthesis of porous NG and bamboo-like NCNTs. The mechanisms of the synthesis process of these carbon nanostructures were investigated by using techniques of SEM, TEM, XRD, Raman and XPS. The porous NG are formed via a self-templated approach by the decomposition and carbonization of the polymer nanoplates. While the formation of NCNTs are due to the presence of FeCl_3_ in the precursor, in which the Fe^3+^ ions were reduced to Fe nanoparticles and act as catalysis for the growth of CNTs. The obtained nanocarbons exhibit very unique structure with many attractive properties such as large surface area, well-organized porosity and abundant introduced N atoms. The results demonstrate the excellent ORR activities of both the NG and NCNTs. The achievement of high performance in electroctalysis by these nanocarbons could be due to their unique nanostructre and properties. And these unique structured nanocarbons may be extend to applied in areas of adsorbents, energy storage and conversion and catalysts.

## Materials and Methods

### Fabrication of the NG and NCNTs

The raw materials (denoted as PEI-MCA) were synthesized via a facile solution approach by assembling of melamine (M), cyanuric acid (CA) and polyethylenimine (PEI) in aqueous solution (details see in the Supporting Information). For the preparation of NG, the PEI-MCA nanoplates were directly used as precursor and subjected to carburization at 800 °C under N_2_ atmosphere. The resultant black product is denoted as NG-800. For the preparation of NCNTs, the mixture of PEI-MCA and FeCl_3_ were grinded into fine powder, and then subjected to carburnization at various temperatures by using the same method.

### Materials Characterization

The wide-angle XRD pattern of the PEI-MCA polymer was recorded on a home-built SAXS machine with 2D detector. The XRD pattern of the NCNTs-800 was carried out at Philips X’Pert Pro Super X–ray diffractometer (Cu Kα source, λ = 1.54178 Å). Transmission electron microscopy (TEM) (JEOL-2010) and field emission scanning electron microscopy (FE-SEM) (JEOL JSM-6330F) were applied to observe the structure of the PEI-MCA, NG and NCNTs. High resolution TEM (HRTEM) and high-angle annular dark-field scanning TEM (HAADF-STEM), were performed on a JEOL ARM200F field emission transmission electron microscope with accelerating voltage of 200 kV. The Scanning force microscopy (SFM) measurements were performed with a Dimension ICON (Bruker) using tapping mode and by using cantilevers with a spring constant of 2 N/m (Olympus OMCL 240). The background of the topography images were flattened using a first order plane fit. The N content of the NG-800 was determined by an elemental analyzer (Vario EL-III, Elementar, Germany). The X-ray photoelectron spectroscopy (XPS) measurements were carried out by a VG ESCALAB MK II X-ray photoelectron spectrometer. Raman spectra were collected by a LabRAM-HR Confocal Raman Microprobe (Jobin Yvon Instruments, France) equipped with a 514.5 nm argon ion laser. The N_2_ adsorption-desorption measurements were performed on an Micromeritics ASAP 2020 surface area analyzer at 77 K.

### Electrochemical Measurements

All the electrochemical experiments were carried out on a CHI 660D computer-controlled potentiostat. Three-electrode electrochemical cell equipped with a glassy carbon (GC) electrode, a platinum counter electrode and a Ag/AgCl reference electrode was applied for the electrochemical measurements. The GC electrode was polished and washed before the measurements. The cyclic voltammetry (CV), rotating disk electrode (RDE) and rotating ring-disk electrode (RRDE) measurements were conducted to determine the catalytic activity of the samples for ORR. Details about the preparation of the catalyst ink and the electrochemical measurements are given by previous reports^23^. The electron transfer number (n) was calculated on account of RRDE measurement by equation^24^: n = 4 ID/(ID + IR/N); The H_2_O_2_ yield percentage (H_2_O_2_%) was calculated based on the equation: H_2_O_2_% = 200 IR/N/(ID + IR/N); where (ID) is the disk current (IR) is the ring current and N = 0.36 is the current collection efficiency of Pt ring.

## Additional Information

**How to cite this article**: Wu, X.-L. *et al*. Self-templated synthesis of novel carbon nanoarchitectures for efficient electrocatalysis. *Sci. Rep*. **6**, 28049; doi: 10.1038/srep28049 (2016).

## Supplementary Material

Supplementary Information
