# Supplementary material for: Self-templated synthesis of novel carbon nanoarchitectures for efficient electrocatalysis
Source: Sci Rep. 2016 Jun 15;6:28049. doi: 10.1038/srep28049 (PMC4908410; doi:10.1038/srep28049)
Supplement: Supplementary Information [file srep28049-s1.pdf]

Supporting Information on

Self-templated synthesis of novel carbon nanoarchitectures for efficient electrocatalysis

Xi-Lin Wu[1,2,4,5], Tao Wen[2,3], Hong-Li Guo[1], Shoujie Liu[1], Xiangke Wang[2,3]*, An-Wu Xu[1]* & Markus Mezger[5]

[1]School of Nuclear Science and Technology, Division of Nanomaterials & Chemistry, University of Science and Technology of China, Hefei 230026, PR China.

[2]School of Environment and Chemical Engineering, North China Electric Power University, Beijing 102206, China.

[3]Key Laboratory of Novel Thin Film Solar Cells, Institute of Plasma Physics, Chinese Academy of Sciences, Hefei, 230031, P.R. China.

[4]College of Geography and Environmental Science, Zhejiang Normal University, Jinhua, 321004, China.

[5]Max Plank Institute for Polymer Research, Ackermannweg 10, 55128, Mainz, Germany.

Correspondence and requests for materials should be addressed to X.W. (xkwang@ipp.ac.cn) or A.-W.X. (anwuxu@ustc.edu.cn).

1. Synthesis and characterization of the precursor (PEI-MCA nanoplates)

All the chemicals used in the experiments were purchased in analytic purity and used without any further purification. Melamine (M) was purchased from Sinopharm Chemical Reagent Co., Ltd.. Cyanuric acid (CA) was purchased from TCI (Shanghai, China). Branched polyethylenimine (PEI, MW 1800) was purchased from Aladdin Reagent Co. Ltd.. For the preparation of the PEI-MCA nanoplates, 0.52 g (4 mmol) of melamine was first sonicated in 20 mL of water in a flask and desire amount of PEI was added in the solution. After the melamine was totally dissolved, 0.32 g (2.5 mmol) of CA with 20 mL water was sonicated to form a suspension and added to the above solution. Immediately, the mixture turned into a milk. The mixture was sonicated for 10 minutes and then stirred for 4 h at room temperature. The obtained milk-like colloids were filtered through a 0.22 μm filter and dried under vacuum at 60 ^{o}C. The dried polymer (PEI-MCA) was further applied as precursor for the preparation of NG and NCNTs. For contrast experiments, 0.52 g (4 mmol) of M and 0.32 g (2.5 mmol) of CA was sonicated in 20 mL of water for 10 min, respectively, and then mixed together to form a self-assmbled polymer (denoted as MCA)

The morphology and structure of the PEI-MCA polymer was investigated by scanning electron microscopy (SEM), transmission electron microscopy (TEM) and scanning force microscopy (SFM). SEM image of the obtained PEI-MCA polymer is shown in Figure S6, in which nanoplates with irregular shape stacking together are observed. TEM image (Figure S7) showed that the size and shape of the nanoplates is inequality. Scanning force microscopy (SFM) measurement (Figure S8) also confirmed the plate-like structure of PEI-MCA, the thickness of one of the nanoplate that stands perpendicular to the surface was measured to be about 25 nm. Wide-angle XRD pattern of the PEI-MCA nanoplates are shown in Figure S9. The characterized peaks at 10.71 °, 18.51 °, 21.43 ° and 27.93 °, corresponding to the (100), (110) (200) and (002) planes, which are well fitted with those of MCA reported in previous reports[1]. The results demonstrated that the structure of the PEI-MCA are

mainly composed of the assembled melamine and cyanuric acid. The PEI polymer may adsorbed on the surface of the self-assembled MCA, which lead to the formation of the PEI-MCA nanoplates.

2. Synthesis of the NG and NCNTs

For the preparation of NG the PEI-MCA powder was put into a porcelain combustion boat and subject to calcination under N_2 flow. The powder was first heated at 550 ^{o}C for 2 h with heating rate of 2.3 ^{o}C per minute, stepped by increasing the temperature to various temperatures (700, 800 and 900 ^{o}C) with the same heating rate and kept at this temperature for 1 h, and then the product was cooled to room temperature. The samples obtained at temperatures of 700, 800 and 900 ^{o}C are denoted as PEIG-700, PEIG-800 and PEIG-900, respectively. For the preparation of NCNTs, 1.25 g of the PEI-MCA powder was first mixed with 0.1 g $FeCl_3$ and grinded into fine powder. The mixture powder was subject to carburnization at various temperatures by using the same method. The product obtained at temperature of 700, 800 and 900 ^{o}C are denoted as NCNTs-700, NCNTs-800 and NCNTs-900, respectively. For further electrochemical measurements, the obtained NCNTs-700, NCNTs-800 and NCNTs-900 were ultrasonically etched in 6 M hydrochloride acid (HCl) for 8h to remove inactive iron species and then filtered through a 0.22 μm filter. The final product was washed to neutral and dried under vacuum at 60 ^{o}C.

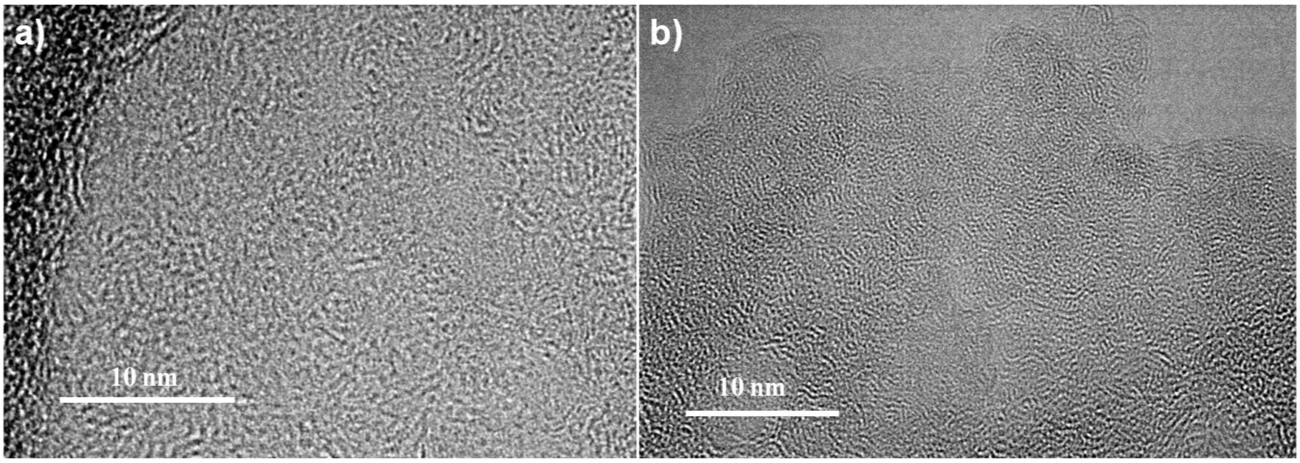

Figure S1. HR-TEM image of the NG-800 a) at the middle and b) at the edge.

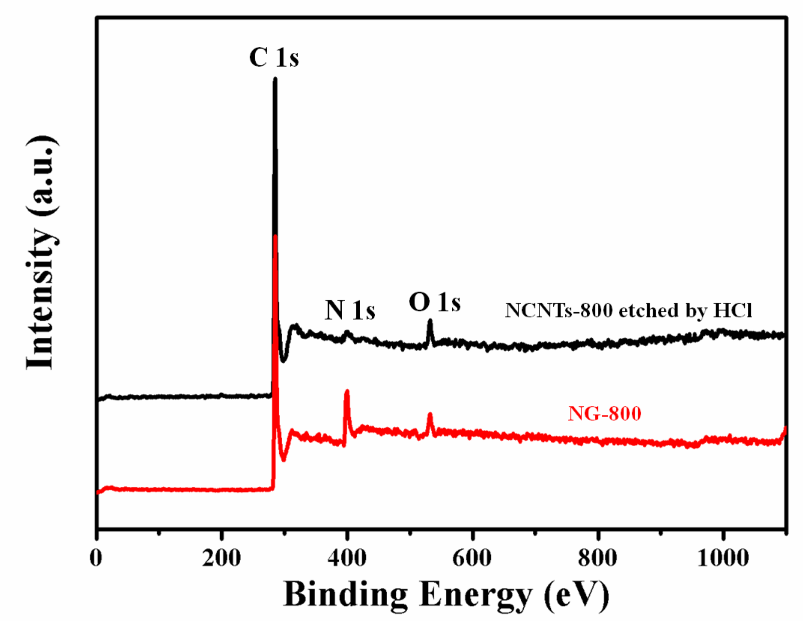

Figure S2. XPS survey spectra of the NG-800 and NCNTs-800 etched by 6 M HCl.

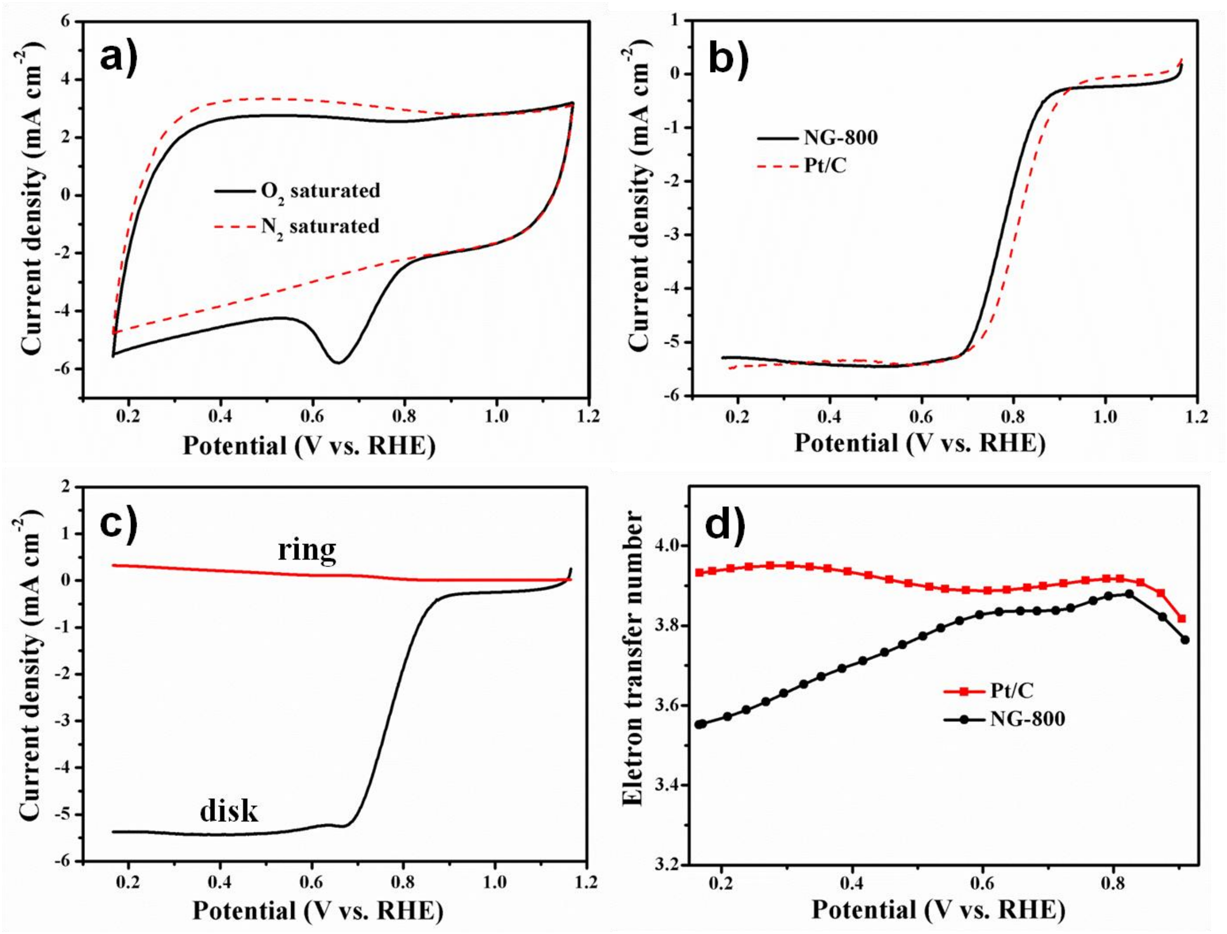

Figure S3. a) Cyclic voltammograms (CV) of the NG-800 in O_2 and N_2 saturated 0.1 M KOH solution. b) Rotating disk electrode (RDE) voltammograms of the NG-800 and Pt/C in O_2 saturated 0.1 M KOH solution, c) rotating ring—disk electrode (RRDE) voltammograms of the NG-800 in O_2 saturated 0.1 M KOH and d) the corresponding electron transfer number.

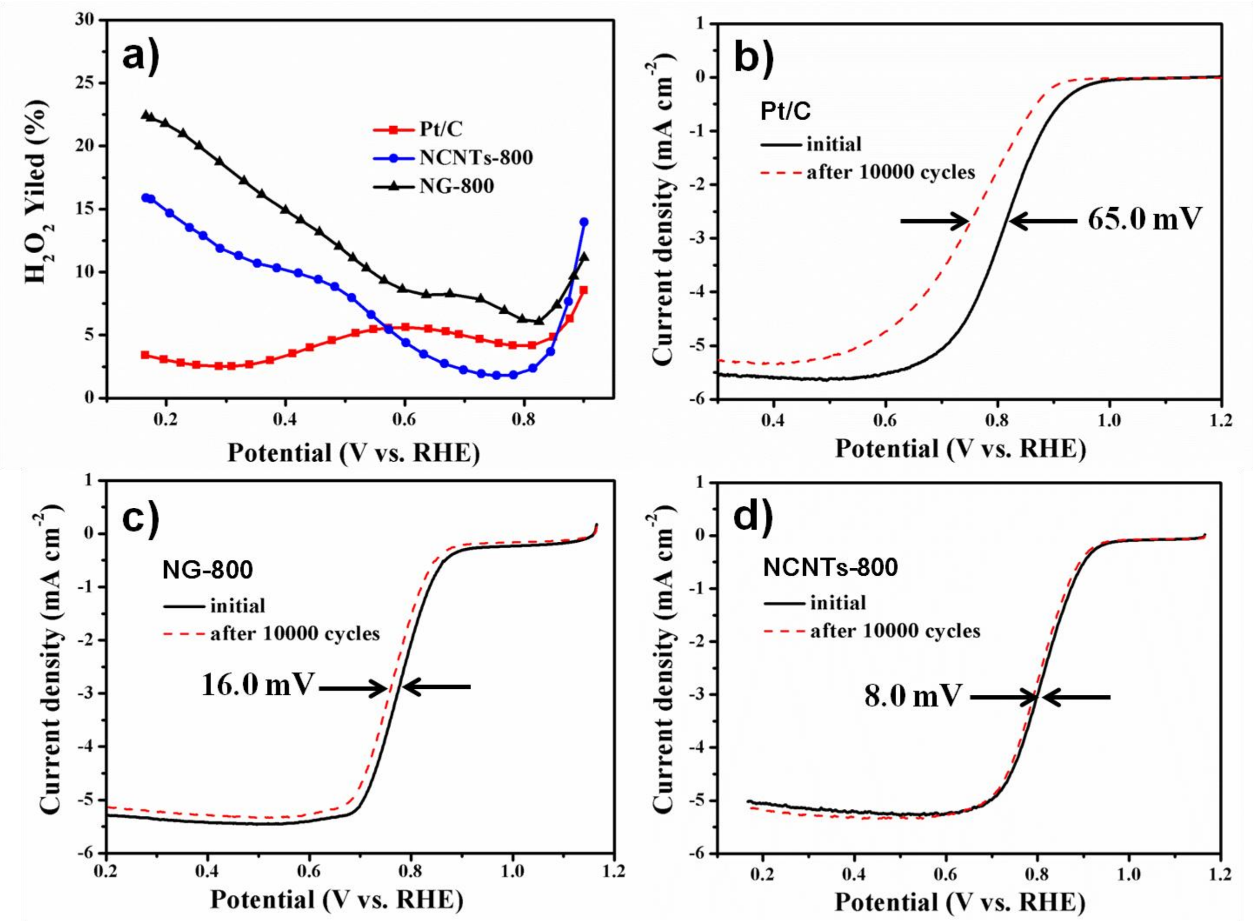

Figure S4. H_2O_2 yield of the NG-800, NG-800 and Pt/C in O_2 saturated 0.1 M KOH solution, Rotating disk electrode (RDE) voltammograms of the b) Pt/C, c) NG-800 and d) NCNTs-800 before and after 10 000 potential cycles in O_2 saturated 0.1 M KOH solution.

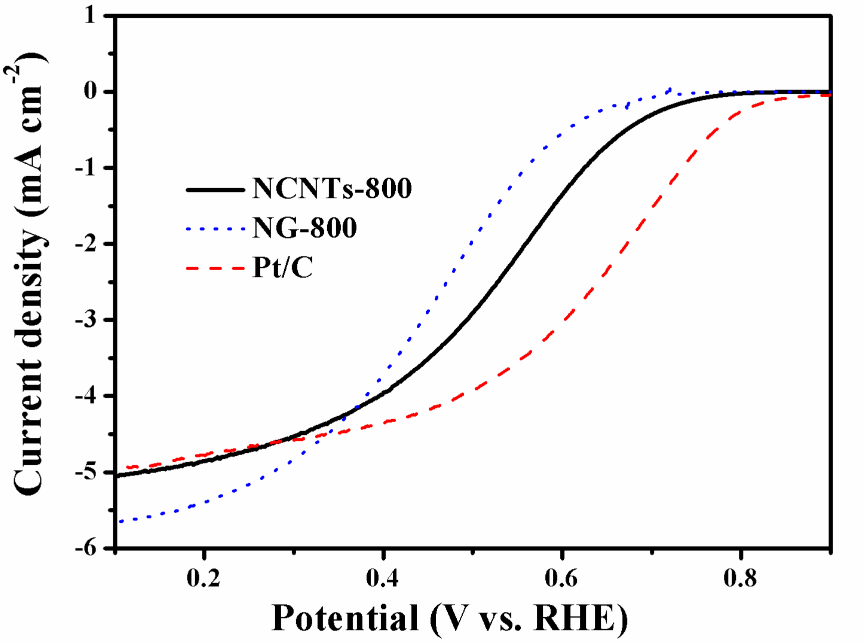

Figure S5. RDE voltammograms of the Pt/C, NG-800 and NCNTs-800 in O_2 saturated 0.1 M $HClO_4$ solution.

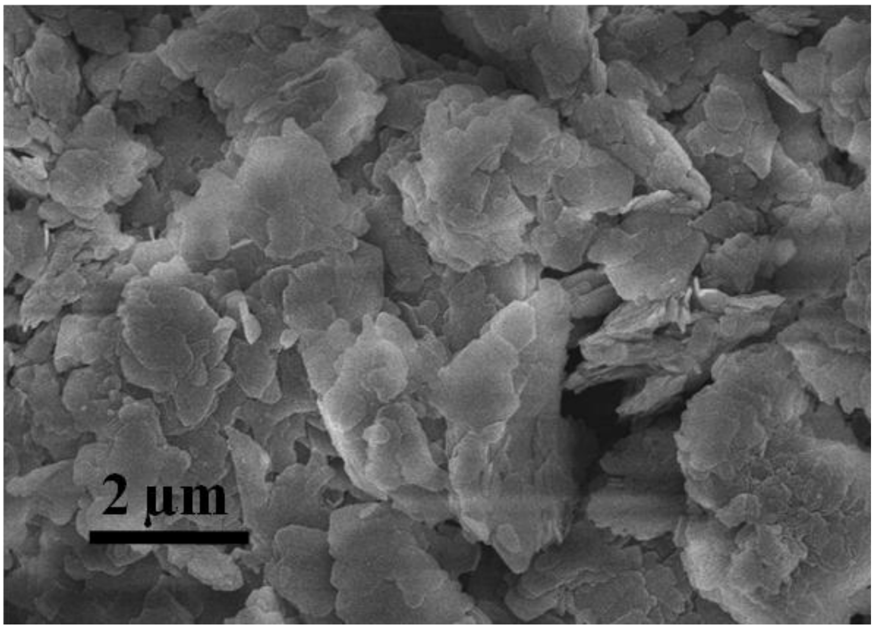

Figure S6. SEM image of the PEI-MCA nanoplates

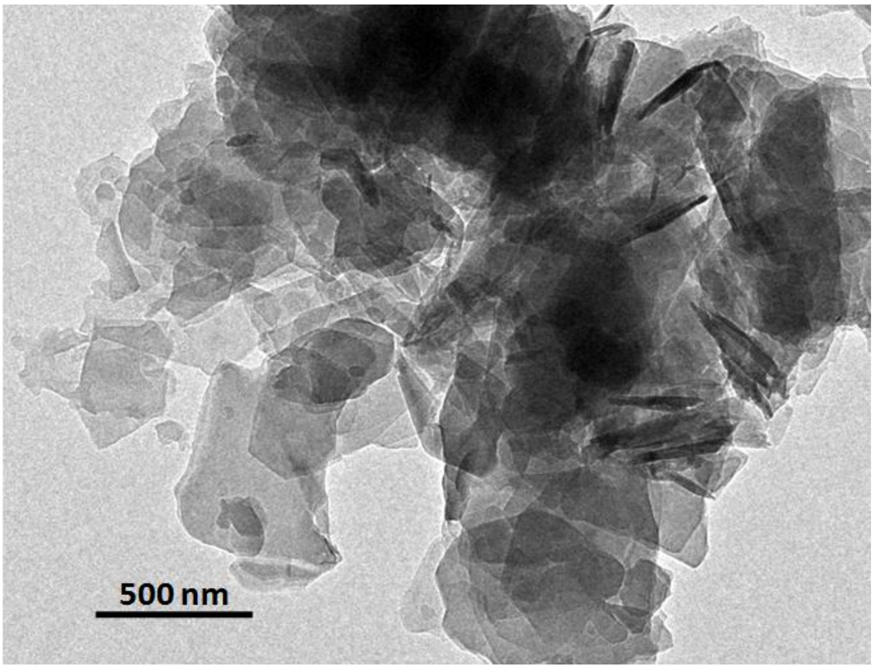

Figure S7. TEM image of the PEI-MCA nanoplates

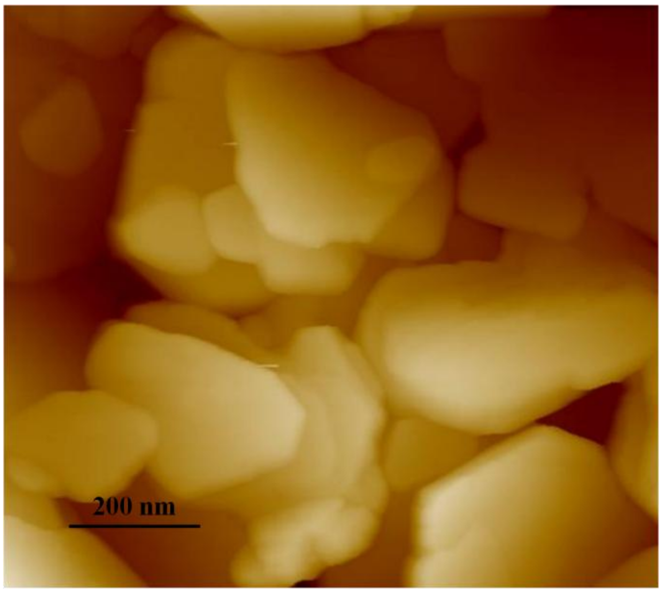

Figure S8. SFM image of the PEI-MCA nanoplates

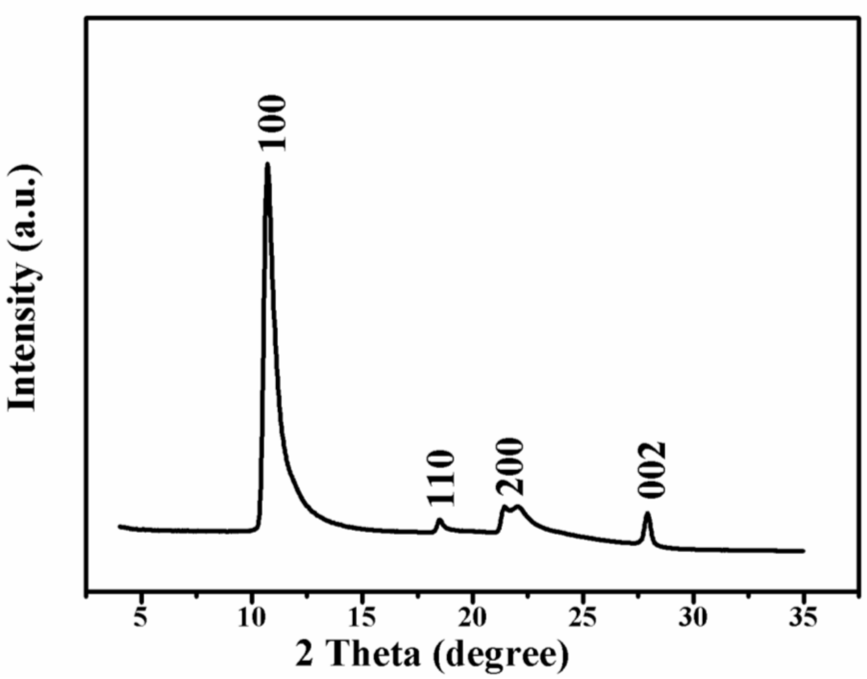

Figure S9. Wide-angle XRD pattern of the PEI-MCA nanoplates.

References

1. Jun, Y.-S. et al. From melamine-cyanuric acid supramolecular aggregates to carbon nitride hollow spheres. *Adv. Funct. Mater.* **23**, 3661–3667(**2013**).
